# Blockchained Industry Information Handoff Based on Internet of Things Devices with Intelligent Customized Object Recognition

**DOI:** 10.3390/s22062312

**Published:** 2022-03-17

**Authors:** Ming-Shen Jian, Chin-Ju Pan

**Affiliations:** 1 Department of Computer Science and Information Engineering, National Formosa University, No. 64, Wunhua Rd., Huwei Township, Yunlin 632, Taiwan; 2 Department of Computer Science and Engineering, National Quemoy University, No. 1, University Rd., Jinning Township, Kinmen 892, Taiwan; cjpan@nqu.edu.tw

**Keywords:** Internet of Things (IoT), blockchain, artificial intelligence (AI), object detection, object recognition

## Abstract

To determine the quality and safety of each product used in manufacturing, the exchange of measured data between machines, operators, production lines, and manufacturing companies is crucial. In this study, we developed a system with customized object recognition capability for the secure blockchain-based transfer of industry information through Internet of Things (IoT) devices. In the proposed system, product history data are transferred through blockchains through artificial intelligence (AI)-based object recognition. Individual objects are recognized and represented using a unique number sequence for use as a private key on a blockchain. The data history can be automatically secured, and all the data are traceable and trackable. The reliability and validity of the proposed system were verified using the Jetson Nano Developer Kit. The proposed AI-based system is a low-cost embedded system. Based on the open-source cloud computing platform, the required computing resources for blockchain computing and storage are available. In an experiment, the proposed system achieved >99% accuracy within 1 s. Furthermore, the computational cost of the proposed system was 10% that of traditional AI systems. The proposed device can be rapidly connected to IoT devices that require limited manual operation and can be adopted in manufacturing and production lines.

## 1. Introduction

Product history information is valuable for customers because it helps them evaluate the safety and quality of products [1,2]. For suppliers, such information helps them maintain the quality and quantity of production and is exchanged at various levels of the supply chain [3]. To obtain complete product history for various products, including food products, raw materials, agricultural products, and electronic products, relevant information must be obtained from various types of production lines and manufacturers. Collecting such information with high accuracy is crucial in smart manufacturing.

Product information is added and modified at different levels of the supply chain, and information falsification may occur during data exchange. Therefore, verifying the accuracy of product data is crucial.

Various algorithms and applications have been developed to ensure data security by leveraging computational resources. Some systems secure information or data by using common security keys, such as the data encryption standard (DES) or advanced encryption standard (AES). With these standards, the data transmitter and receiver can exchange data through the same security key for encryption and decryption [4]. However, the safety of a common security key may be compromised after long-term use. Therefore, an asymmetric system comprising a public key and private key was proposed. Transmitted data can be secured using a private key and decrypted using the corresponding public key [5]. In addition to the security of data transmission, the undeniability of content should be considered. The content or data exchanged in different steps of the data chain should be undeniable and reliable. Thus, all inspection reports from the first step to the last step of the inspection chain should be verified as reliable.

An undeniable inspection report should include information related to the date, machines, inspectors, data receiver, and data sender. Similar to online transactions, all digital information related to information exchange should be recorded and verified. Bitcoin [6] is a famous network for online transactions. The reliability of online transactions can be verified through complex computations by using blockchain technology [7,8] with a private key and distributed records. Therefore, except in the case of 51% attacks, the reports or transactions included in a block can be considered undeniable.

When implementing secure information transfer, one must consider that small- or medium-sized factories can only hire a few operators with limited knowledge and use limited network technologies. In addition, these factories have limited budgets for upgrading their traditional product lines. Blockchain-based technologies can be adopted to achieve secure information transfer, tracing, and tracking at a low cost in the manufacturing industry. Internet of Things (IoT) devices should be used for accurately collecting as much relevant information as possible from the environment or product line. To ensure the correctness of the information exchanged by manufacturing companies or in the supply chain, we propose an artificial intelligence (AI)-based system with customized object recognition capabilities for the blockchain-based transfer of information through IoT devices in industrial applications. The contributions of this paper are as follows:

1. In the developed AI-based system, each customized object, operator face, or trade item is identified as a unique object. Because blockchain application requires the use of a unique private key, the features of the image recognition corresponding to object or face based on the AI system can be the available solution. Thus, information on individual objects can be connected to blockchain by using the private key of these objects.
2. The proposed system can be connected to IoT devices (such as cameras)linked to a wireless or wired network. Moreover, an intelligent algorithm can be programmed into the IoT devices used in the proposed system for object recognition along the production line. IoT devices enable automatic information exchange. The computational cost of the proposed system is 10% that of traditional AI systems. Smart IoT devices can directly recognize local objects; thus, object recognition can be achieved locally with relatively low network transmission. Only the corresponding data and related private key for blockchain computations on cloud are required.
3. All the industrial information exchanged through blockchain is undeniable. Information can also be stored on a cloud for tracing and tracking. When information is transferred between supply chain members or between production lines and workstations, the identified object should be the main private key for the blockchain. The data of an object with the corresponding private key can be traced and tracked, and data cannot be traced using a wrong key.

The rest of this paper is organized as follows. Section 2 details studies related to the present research. Section 3 describes the proposed system and data collection procedure. Section 4 presents the results of this study. Section 5 presents a discussion on the obtained results, and Section 6 concludes this study.

## 2. Related Studies

Cloud computing, which is based on virtualization technology, was developed for conducting complex computations requiring massive computation resources [9]. Physical computational resources, such as processors, memory, and storage, can be virtualized and merged as computation resource pools. In virtualized technology, the resources used for computation vary with the virtual computing task. Through the cloud network, remote users or systems can obtain the computational resources or services required. In addition to virtual machines, Docker containers [10] are popular in cloud computing. A Docker container is established on the basis of the Cloud Docker environment, and such a container can be used frequently after its establishment. Services or programs can be included in a Docker container in advance. By using the same Docker engine with virtualization technologies, one can implement various containers on different computing nodes (computers). Thus, Docker containers can be used for rapidly providing the same services or programs at different computing nodes.

Studies have investigated the security frameworks of IoT services. A new IoT framework based on cognitive systems and blockchain technology can provide more benefits relative to existing IoT frameworks [11]. In an IoT network environment, data transmission verification is performed through instruction detection based on a blockchain to ensure data security. Security messages provided by IoT devices can indicate the presence of external malicious instructions [12]. By incorporating blockchain technologies into privacy-based applications, personal information can be secured. In addition, smart contacts based on a blockchain can be implemented in online services and commerce [13]. However, in the case of such contracts, the security of data exchange through the online network as well as the reliability and accuracy of the data transmitted between the transmitter and the receiver should be considered.

To achieve more functional IoT services, Artificial Intelligence of Things (AIoT) was proposed [14]. By using IoT devices with the functions of edge computing and cloud computing, AI applications more suitable to the local IoT environment can be developed. Social computing applications can be used to provide personalized services for groups [15]. An IoT platform that ensures privacy and security is urgently required. In addition, to ensure the security of IoT applications, AI can be used to detect serious IoT security threats, such as Denial of Service (DDoS) attacks, device authentication, and intrusion detection. However, a security algorithm for IoT applications can cause negative effects [16]. Therefore, when developing AI-based services to ensure the security of IoT applications, the computing ability and computing performance of the IoT environment should be considered.

Various algorithms and methods have been developed to ensure secure data exchange. If a security method is insufficiently strong, a malicious third party can breach the security and access confidential data [17]. The Electronic Frontier Foundation (EEF) organized and funded a project to develop the EFF DES cracker for decryption. This cracker can breach the DES through key cracking within limited time [18]. Therefore, in the present study, a blockchain-based security method was used instead of the tradition symmetric key security method.

Blockchain technology is used for ensuring distributed information security [19,20]. It can be used to decentralize a database. Each computing node of the blockchain operates according to a consensus algorithm, such as the proof-of-work (POW) algorithm. Through cryptography, all information records, which are also called blocks, can be chained to or linked with each other. Each block includes the hash code of the previously chained block, the timestamp, and the transmitted data. The distributed computing can be used to ensure that the recorded block is unalterable. Thus, blockchain participants can independently verify a data transaction.

When a transaction (data exchange) occurs, detailed transaction data are secured according to the asymmetric cryptosystem or digital signature. By using the hash function, these secured transacted data are then represented as a unique hash code. This unique hash code is broadcasted to the other computing nodes of the blockchain network. To enhance the security of and manage transmitted data, the blockchain transaction or intelligent contract is used in different supply chain applications [21,22]. The transaction can be secured and traced, and the stored data can be analyzed.

After receiving one or more non verified hash codes, a computing node places these hash codes into a block; thus, one block may include many transactions. The computing nodes that receive the hash code verify the block according to the POW algorithm. The fastest node to complete the calculation of the POW algorithm can verify the block. The verified block is subsequently broadcasted to the other nodes of the blockchain network. The other nodes of the blockchain network validate the received block. If the nodes can confirm the validation of the transaction block with a valid digital signature, this block is connected to the blockchain (or chained). Finally, this chained block becomes undeniable and unalterable.

Blockchain systems with distributed watchdogs have been proposed to protect software supply chains [23]. By assigning each entity a unique ID, a dual identity is implemented for each entity in the blockchain and IoT network. In addition, through outlier detection, specific malicious activities can be identified [24]. By integrating blockchain and IoT systems, a transparent, flexible, and secure data transmission system can be achieved for a supply chain [25]. Some studies have integrated blockchains with machine-learning-based AI methods for managing blockchain data [26]. However, because the POW algorithm is used, blockchain computations are computationally very expensive. Thus, methods for supporting blockchain computations are crucial. IoT devices are popular and widely applied [27,28]. Most of the IoT devices used in the reviewed literature use wireless networks for data transmission [28,29]. In manufacturing or production lines, the use of numerous IoT devices results in massive quantities of data being transmitted through the network. To reduce the data transmission load, the data of local IoT devices must be managed in advance. Because of the limited computation resources of IoT devices, their data must be managed using different devices and layers. Thus, applications involving complex computations, such as AI applications, cannot be easily executed on IoT devices.

Embedded systems with portable sensors have been incorporated into intelligent IoT devices. If sufficient computation resources are available, embedded systems can not only collect environmental data through sensors but also provide intelligent services, such as object detection, face or object recognition, and AI computing [30]. For object recognition or detection, image processing can be performed by a local embedded system without the entire image being transmitted through the network; thus, the size of transmitted data can be reduced. Therefore, in this research, the traditional IoT network and structure are modified to develop advanced IoT devices.

## 3. Proposed System

**Figure 1:**
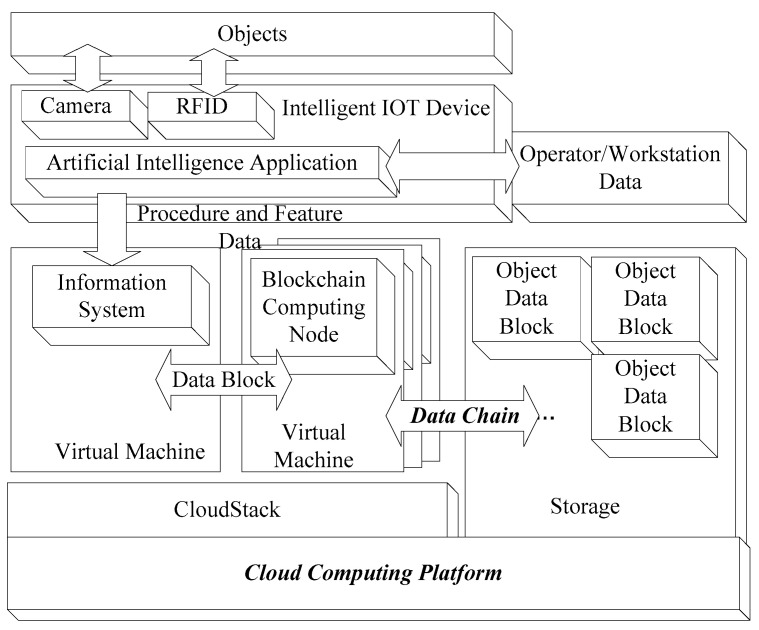
Structure of the proposed intelligent Internet of Things (IoT) device with a cloud computing platform for smart manufacturing.

Adapted to the implementation environment, the proposed system is divided into three parts: an intelligent IoT device; a blockchain computation block, which uses the blockchain data node and computing node based on cloud environment; and a data exchange block (Figure 1). If an embedded system can provide sufficient computation resources, traditional AI services can be removed from the remote server and provided by local IoT devices. Thus, AI-based services, such as object detection, face or object recognition, and production line management, can be implemented directly by a local embedded IoT device [31]. Only the sequential data of detection or recognition results, rather than entire images, are transferred to the remote server or cloud for further processing. Local intelligent IoT services can filter out large quantities of data and reduce the data size. Therefore, the proposed intelligent IoT system can serve as a local information gateway and handle information from connected sensors. In this research, intelligent IoT devices are used for edge computing. However, to provide storage space and execute blockchain computations, additional computation resources are required. Therefore, the remote information system on the cloud receives data from intelligent IoT devices locally to conduct additional blockchain computations. Because the blockchain operates according to the POW algorithm, multiple virtual machines are established as blockchain computing nodes on demand. Finally, the blocked and chained data can be stored in the cloud. Figure 1 illustrates the relationship between objects, intelligent IoT devices, and blockchain services on the cloud. No additional hardware or gateway is required in the structure depicted in Figure 1; thus, the deployment cost of this structure is relatively low.

To further reduce the network traffic, the gateway and fog layer can be used for preprocessing data. The intelligent IoT system developed in this research satisfies the computing requirements of a traditional production line. In addition, this system, which is an embedded system, can be connected to various sensors [31]. Since the intelligent IoT system in this research could provide enough computing resources, to reduce the cost of upgrading production lines, the additional gateway and fog layer are not used in this research.

### 3.1. Intelligent IoT Device

Suppose that a network can be accessed by each IoT device. Images or videos captured using a camera can be transmitted continually through such a network. Considering the implementation environment, this study selects an embedded system with the network and camera. Depending on the specific manufacturing requirements, such a system may possess single-photo or continuous-frame streaming functionality. According to the hardware of an IoT embedded system, object identification can be executed locally or on the cloud. Moreover, because a production line may have different types of products or objects with custom specifications, different types of intelligent systems are available for IoT devices. In this research, two functions are considered: remote object recognition and local-side recognition. When numerous customized objects with considerable differences and simple appearances must be recognized, either the remote object recognition or local-side recognition functions is used. If the appearances of objects are similar but popular with huge available data, the local-side recognition function is selected; otherwise, the remote recognition function is used.

The central processing unit (CPU), graphics processing unit (GPU), memory units, and input/output ports of currently available embedded systems are much better than the traditional Arduino embedded system. The software of embedded systems includes an operation system. Therefore, an embedded system can execute programs or applications. Consequently, intelligent IoT devices can provide responses according to the requirements of the remote manager or program. Furthermore, intelligent IoT devices can be upgraded by providing them with updated or new functions. Therefore, numerous AI-based functions and services are currently available for intelligent IoT devices. Arduino-based IoT devices have a computing power of approximately 16 million instructions per second and a limited processing speed. Current embedded systems have a clock speed of more than 700 MHz. Some embedded systems contain 1.4 GHz CPU with a GPU and memory units. The intelligent IoT system developed in this system is a programmable embedded system that can be connected to various sensors.

### 3.2. AI Applications

The objects used in manufacturing should be identified. When a customized object is at the product line or a workstation before being transferred from one production phase to the next, a local IoT device with one camera or two cameras is used to capture a photo or video of the object. Considering the possible required information, one or two cameras are connected to an IoT device to obtain images from different perspectives. At least one check point exists after every production process or workstation.

By using AI algorithms in intelligent IoT devices, image classification and object localization can be conducted for smart manufacturing. Image classification and object localization are performed to identify individual objects. Various products can be produced by a single manufacturer. Different product lines produce different products, and the products from the same product line are similar. Therefore, considerable data related to general or normal products are obtained as training data. The binary cross-entropy loss function is used to classify training data. Because the differences between normal and customized objects would be similar, these objects cannot be suitably classified using the softmax method. Similar objects can be included in a complex data set with nested relationship labels. Data relationships can then be determined through multilabel classification.

Furthermore, because of the limitations or functions of a production process or workstation, the size, appearance, and shape of a customized object are only marginally different from those of a normal object. Therefore, the K-means algorithm must be used on the training data. According to the existing data on manufactured products, a suitable number of object features can be obtained on demand. On the basis of these features, normal objects can be differentiated from customized objects. Finally, feature information and the results of K-means clustering can be used to identify and classify the objects located at a check point. Thus, AI algorithms can not only classify and locate objects but also distinguish them according to factors such as their color, appearance, shape, and size. Customized objects can be represented as a sequence of numbers corresponding to the detected features.

An embedded system with a GPU, camera, and network connection can provide AI functions and services. Intelligent algorithms and suitable software applications can be incorporated into an embedded system, which transfers local identification results to a remote server. By contrast, IoT devices with a camera and network connection but insufficient computation resources transfer entire photo or video frames to the remote server for object identification. In this research, the proposed intelligent IoT system is implemented as a local computing device for providing AI-based services. Thus, the amount of data transmitted through the network can be reduced.

In this research, object detection and face or object recognition are executed using intelligent IoT devices. The image captured by a camera module connected to a network can be analyzed locally. The AI method for detection and recognition is implemented using a neural network deployed through TensorRT. However, considering the training data and training workload, training can be conducted in a cloud environment. The trained AI application can be packaged as the container of the Cloud Docker. In addition, the recognized training objects or faces are represented using sequential numbers corresponding to specific features. When a camera or sensor detects an object, it captures a static image of the object, and its AI application analyzes the captured image. Different types of deep learning architectures exist. In this research, GoogLeNet with a 22-layer network is used. AI services can be used to detect and recognize objects or faces according to the features of these objects and faces. Thus, an AI-based IoT device can collect the following information: the sequential number corresponding to a recognized object or face, the location, the time, and the date.

### 3.3. Blockchain Application

After identification at the local side during the production process, the remote server receives the number sequences corresponding to the detected features of objects. Because object data must be exchanged between different phases, procedures, workstations, or supply chain members, the accuracy of the transmitted data must be verified. Suppose that a data transmitter and data receiver are exchanging data. Instead of using receipts acknowledgement, the transmitter and receiver can use individual identification information or codes for data transfer. Data exchange is considered a transaction. When data must be transmitted, the transmitter, which can be an operator or IoT device, sends a request to the receiver. If the receiver is available, it sends a transaction query that includes the required information related to it to the transmitter. When receiving the transaction query, the transmitter begins to block object data from a workstation or product line and an intelligent IoT device according to the defined blockchain algorithm and transmitter information (or private key). Thus, on the basis of the blockchain technology, the aforementioned data transaction can be secured using a private key, and the data transmission system is similar to an asymmetric cryptosystem. After the computation and verification by other computing nodes, the data transaction between the transmitter and the receiver is secured as a “block” and undeniable.

In this research, the number sequence corresponding to an object is used as a key. This sequence varies with the object, location of the IoT device, and time. In blockchain-based applications, data blocks related to each other are chained to each other. Therefore, to obtain a final summary of the data, all the data from different procedures, workstations, and operators are chained together. Finally, all the data presented in the final production history are trusted and undeniable.

**Figure 2:**
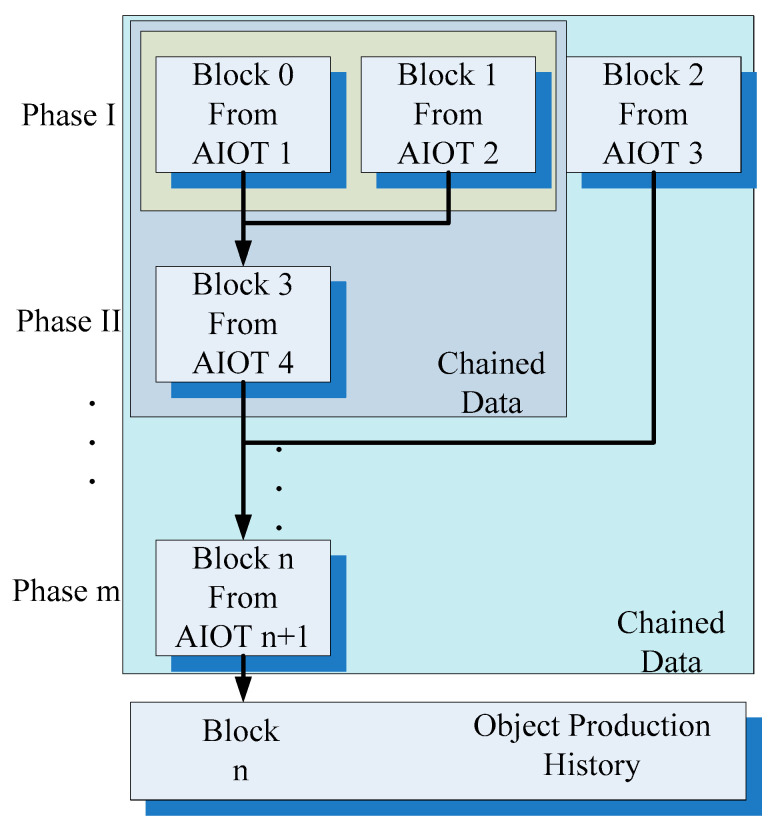
Proposed system for the transfer of blockchain information through IoT devices.

Figure 2 presents the concept of the proposed system and service. Because various production processes or workstation phases may be involved in manufacturing, multiple check points should exist between two workstations, operators, or production lines. Suppose that AIoT devices are located at the aforementioned check points. Before an object is transferred from one production phase to another, the object is checked and identified by a local AIoT device. After checking, each AIoT device transfers the data collected by it, including the object features and other information related to the production history, to a remote server. The blockchain computing nodes in the cloud block all information by using the private key of object features, which indicates the number sequence corresponding to the object. Depending on the production line and processes, data related to one object must be integrated from various blocks. Therefore, the information blocks obtained from different AIoT devices are combined and chained as a new block containing additional information related to a new production phase. For example, as shown in Figure 2, block 0 and block 1 are chained to a new block, namely, block 3, in a new production phase by AIoT device 4. If *n* + 1 AIoT devices are used in *m* production phases, *n* chained information blocks are generated because each AIoT device creates a new data block. Finally, the last block indicates that the data exchanged between the AIoT devices (or different production phases or workstations) are undeniable. Thus, the data in the last block could be used as the valid production history of the final object.

### 3.4. Cloud Computing

Blockchain computations are computationally expensive and are thus suited to be offloaded to the cloud. A cloud platform can host multiple virtual machines with the same configuration for executing the POW algorithm. Therefore, open-source cloud computing is adopted in this research. The proposed blockchain-based process and data storage can be implemented by the Docker container. Based on the cloud platform, multiple virtual machines can be created as the blockchain computing nodes for POW services. Because the configuration and programs of virtual machines can be formatted as ISO files, the total number of virtual machines required for executing the POW algorithm can be dynamically changed. Thus, this algorithm can be implemented within an internal network. Furthermore, all the blocked data can be chained and stored on a private cloud.

## 4. Verification and Results

A cloud computing platform based on the Ubuntu open-source operation system was implemented, and a virtual machine was created in a CloudStack environment. To define and differentiate normal and customized objects, the K-means algorithm was used for training data classification. Numerous operators exist in an industrial production line. In this research, human faces of operators with more features were tested. To classify training images into different groups, more than 60 features are used.

**Figure 3:**
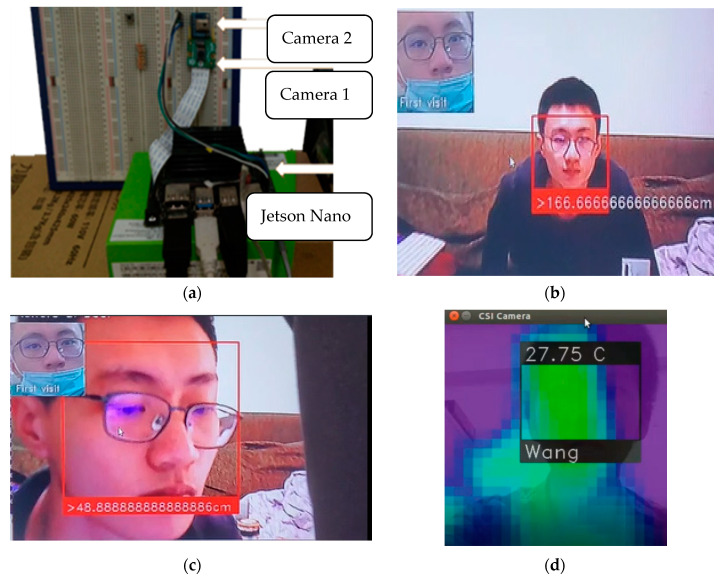
Implementation and verification of Artificial Intelligence of Things devices in the Jetson Nano Developer Kit for artificial-intelligence-based edge computing: (**a**) edge computing hardware, (**b**) facial recognition based on edge computing, (**c**) distance measurement for a recognized face, and (**d**) combined result of temperature and face recognition.

To implement the developed system, the Jetson Nano 4G Developer Kit (NVIDIA) with multiple sensors was used. Because AIoT devices are used for object identification, including image classification and object location detection, the proposed system required a high computation ability. Figure 3a illustrates the implementation of the AIoT devices for edge computing. To provide and verify multiple functions, Raspberry Pi Camera Module V2.1 [Camera 1 in Figure 3a] and an AMG 8831 8 × 8 infrared thermal camera [Camera 2 in Figure 3a] were connected to the Jetson Nano Developer Kit through wire connections. The two cameras could provide different images for detection and recognition by the developed AI applications. Static images from both cameras could be merged into a single image for object or face detection. In addition, because the AI applications were already subjected to training, object and face recognition could be conducted by the local Jetson Nano without transmitting the image to the remote server. The proposed system implemented on Jetson Nano could recognize operator faces. After training, the proposed system could identify a face within 3 s if the total number of photos recorded in the database was less than 20 [Figure 3b]. By suitably editing the adopted program and algorithm, the distance between the recognized operator and the camera could be evaluated [Figure 3c]. In addition, the recognition results of the two cameras could be combined to obtain a single conclusion. Because of the detection distance and accuracy of the AMG 8831 camera, it could not detect the temperature with high accuracy. However, accurate results were obtained when combining the face recognition and temperature detection results [Figure 3d].

Jetson Nano runs at only five Win AI applications when a GPU is used. By using a GPU and CUDA, deep learning can be achieved by Jetson Nano for image recognition and object detection. By using TensorRT, objects can be classified according to the probability of classification. According to the training data and given image data, an identified object can be represented by a number sequence corresponding to the relevant classification group.

**Figure 4:**
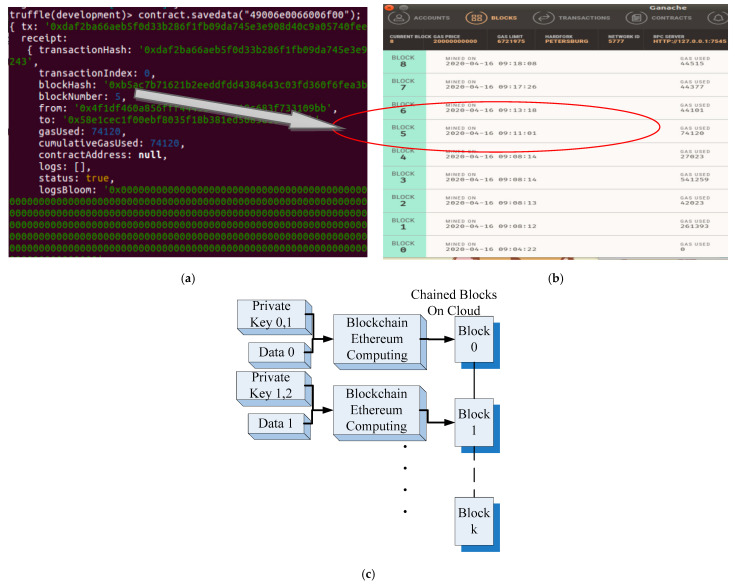
(**a**) Plaintext of the relevant object’s private key. This plaintext comprises the number sequence in TX that is included in the smart contract. (**b**) Blockchain data stored on the cloud platform after blockchain application. (**c**) Abstract concept of the proposed system blocking the plaintext or data by using the relevant private key.

Data are exchanged internally between operators and the proposed system, and ensuring the accuracy of the exchanged data is crucial. When an object is transferred from one check point of the production line or workstation to another, the transmitted data should be blocked according to the blockchain application. Figure 4 presents the data related to the object to be delivered. Figure 4a presents the plaintext of the production data of the object to be delivered. The plaintext comprises a number sequence, which is chained using the contract.savedata() function. In Figure 4, TX presents the face recognition result of the operator. These data are used as the unique private key for blockchain computations. Relevant data are blocked and chained to the cloud. The number of the new block after the chained is five. The two terminals of information transfer are included in the “from” and “to” fields. Related information such as time and file type is included in the smart contract by the proposed system. Figure 4b depicts the blockchain data stored on the cloud platform after blockchain application. Chained data with related information, such as block number, block date, and time, can be presented as web pages. Figure 4c illustrates the abstract concept of the proposed system blocking the plaintext or data by using the private key corresponding to the previous sender and current receiver. When the data (data 0) is exchanged, recognition results of operators or objects at transmitter AIoT and receiver AIoT are used as private keys for blockchain. In other words, the data are secured by private key 0 and private key 1. It also means that both sides agree with the data exchanging which is blocked as the smart contract and numbered as block 0. After processing, there exist new data (data 1) at the current AIoT device which will be exchanged with the further (next) AIoT device. Hence, the current AIoT device as transmitter and the next AIoT device as receiver would also provide the private key, such as private key 1 and private key 2 for blockchain computation. The new data exchanging will be chained as the block 1.

**Figure 5:**
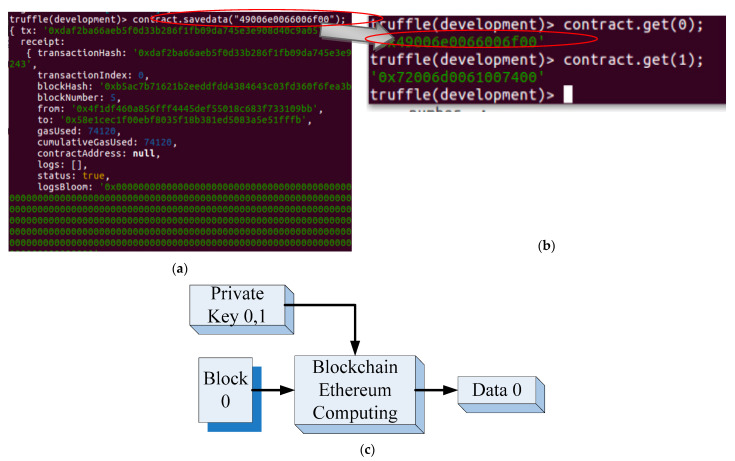
(**a**) Plaintext of an object that is represented in the hexadecimal format as “4900630066006f00” before being chained using the *contract.savedata*() function; (**b**) data retrieved from the block by using the *contract.get*() function, which is the same as the original data; and (**c**) obtaining the original plaintext or data by using the corresponding private key.

In addition to the object’s blockchain data, all the blocked data should be verified. For example, as depicted in Figure 5a, if the number sequence corresponding to specific object data is represented in the hexadecimal format as“4900630066006f00”, the *contract.savedata* (“4900630066006f00”) function chains the data into blocks, which are stored on the cloud as sequential hash-codes; thus, the information included in blockchains is secured. For verifying a block, the *contract.get*() function is used to retrieve the original unchained data in the form of the original plaintext (i.e., “0x4900630066006f00” in Figure 5b). Figure 5c illustrates the feasibility of obtaining the original plaintext or data by using the corresponding private keys of the previous sender and current receiver.

Considering the verifiable block and undeniable object data, the information corresponding to aspects such as individual operators, workstations, and check points can be included in the data chain. If such data are included in the data chain, any wrong data can be tracked and traced to a production processor or operator. In this research, private keys for data blocks were obtained using the unique feature vector of individual objects.

## 5. Discussion

Currently, computer vision and AI applications are widely used. To improve smart manufacturing, data from production lines or processes should be collected and transferred automatically through a traceable and secure data chain. Because of space and power limitations in most factories, the IoT devices used in factories for monitoring and data collection cannot occupy massive spaces and consume considerable power. Moreover, a high computational cost is associated with photo or video streaming through network for further analysis, identification, and classification. In addition, when a high number of IoT devices are used for monitoring and identification, the central server for AI services has a heavy computation workload. Therefore, distributing the computation workload by integrating edge computing with smart manufacturing is crucial.

AI-based facial recognition is computationally expensive. Most facial recognition applications must be implemented using a GPU. According to a report related to Bitcoin mining, a GPU can enhance mining efficiency. All data exchanges or transactions should be verified after executing the POW algorithm. Therefore, the hardware required for blockchain applications consumes considerable computation resources. Considering the implementation of the sensors and IoT devices, only the embedded system with limited computing ability with low cost could be available for development.

Data training and deep learning are crucial aspects related to AI services. A neural network should be deployed in an embedded system for image recognition, object detection, and segmentation. Most embedded systems, such as Arduino, or CPUs, such as Raspberry Pi, have a processing speed of 16 million instructions per second. To execute advanced AI algorithms rapidly, a GPU should be adopted for evolutionary computation and deep learning. Therefore, AI-based services are usually developed and implemented on workstations or a high-level server with a high-end graphics card. In this research, the AI service was limited to finite object recognition; therefore, the proposed system only used 10% of the computation power of the adopted GPU.

In this research, the Jetson Nano embedded system had sufficient computation resources for object classification. Thus, after training, the adopted AI application could be executed on Jetson Nano with relatively low network transmission. Furthermore, this embedded system could be connected to different sensors. Thus, this system could act as an AIoT device for edge computing and reduce the cost of data transmission and the computation workload at the central server. By controlling the total number of features used for classification, most objects could be identified on the basis of the evaluation probability. The Jetson Nano system could identify the faces of 20 operators with an accuracy of higher than 99% within 1 s. It represented features of each object or operator as unique number sequences. The features of the 20 faces in this research were independent and different because no images of twins were captured. The training time for the implemented AI service was short, and some faces could be immediately identified without any pre-training. Thus, the proposed system can be integrated into production lines immediately with limited data training.

By integrating blockchain services for data transmission, blockchain data can be computed by multiple blockchain computing nodes, and the computed data are undeniable. Thus, information or data from the production line or workstation can be secured. In addition, by using an AIoT device, a unique private key can be obtained for a blockchain service. Therefore, the blockchain data corresponding to a specific object can be traced and tracked, and the data stored on a cloud can be chained as the product history. In addition, on the basis of data chains, data errors can be found and assigned to a specific production phase or operator. The proposed system can improve the performance of a production line and the quality of products produced on the line. Because encryption and decryption require computation resources, the embedded system, which has limited computation resources, cannot dynamically change the secure key for encryption and decryption. In addition, to trace and collect information related to product history, a unique key corresponding to an operator or object is required. In this research, all the information related to a specific object or operator could be secured and chained independently. Thus, the product history of an object could be chained according to the production steps or time. The operation performance could also be chained to the data corresponding to an individual operator. In addition, each data block could be used for data tracing and tracking. The data blocks are secured and could only be decrypted using the unique key corresponding to an individual operator or object.

### 5.1. Theoretical Contributions

In the proposed system, blockchain technology is implemented using a unique key corresponding to an individual operator or object. By using the Open Neural Network Exchange Format, different deep learning modules with different training frameworks, such as TensorFlow, Keras, Caffe2, and PyTorch, can be incorporated into the proposed system. After transforming training framework data into the Open Neural Network Exchange Format, TensorRT, which is based on the C++ programming language, can be used to optimize the training framework as an inference engine. A GPU can then be used to obtain recognition results with high performance. In this research, the computation workload was reduced using only 68 facial features, rather than the 128 features of the face module, for evaluation and classification. Thus, by reducing the total number of features considered in the classification, the computation workload can be reduced.

### 5.2. Practical Implications

The incorporation of IoT devices with AI applications into a low-cost embedded system allows these devices to provide more functions and services locally. In the aforementioned case, managers or controllers can still send commands to IoT devices for specific purposes. By reducing the features used for object detection and recognition, the hardware cost for AI computations can be reduced by 90%. In this research, after training, the proposed system could achieve an accuracy of 99% in object or facial recognition within approximately 1 s. Thus, AI applications can be feasibly integrated into the proposed system. When the proposed system used sufficient computing resources, most data were filtered before being sent to the remote server. The IoT devices in this system could not only function independently but also receive commands from a remote controller.

Through blockchain computing, data related to a single object or operator can be chained and secured. The production history is represented as chained data blocks. The manual operation corresponding to individual operators can also be recorded. In addition, because all the blockchain data are stored in a cloud computing environment, they can be tracked and traced to a specific object or operator.

By using the Cloud Docker engine, the Docker container of an AI application can be established and then deployed multiple times indifferent IoT devices. Thus, by training an AI application on a cloud computing platform once, all IoT devices with the same container can be provided the same intelligent functions. Training and packaging in a Docker container can reduce the maintenance cost even when AI applications must be updated or upgraded.

### 5.3. Limitations and Future Research Direction

In this research, a system with sufficient computational resources that integrates IoT devices with AI applications, such as object detection and recognition, was developed. The total cost of the developed system is lower than that of traditional AI computation hardware but higher than that of basic embedded systems, such as Arduino. Future studies can combine multiple inexpensive embedded systems with sensors into one system managed by a single intelligent IoT device.

Future research can also examine the cost of system deployment in numerous manufacturing or production lines and the cost of storing blockchain data in the cloud. The big data corresponding to an object or operator should be analyzed for enhancing management or production performance.

In addition, the PoW consensus algorithm may cause energy wasting. The ASICs and mining pools would also contribute the 51% attack. Therefore, considering the available blockchain platform, the Paxos algorithm or Ethereum with smart contract service based on proof-of-stake would also be possible future works for the AIoT information security.

The proposed system is attractive because it only requires light upgrades of traditional production lines. In practice, only a few operators with limited knowledge and network technologies manage production lines.

## 6. Conclusions

The proposed system can manage the data transmission process and ensure that the transmitted data are undeniable. Thus, the proposed system can be used to verify the accuracy of blockchain data from the first to the last production phase. Blockchain data are accurate, reliable, and secure. The proposed system, which contains intelligent IoT devices with AI applications, can identify objects and human faces when objects are transferred from one production phase to the next. After training, AI applications can be executed on IoT embedded systems. By distributing the computation workload between edge computing and the remote server, the cost for upgrading existing manufacturing lines can be reduced.

The experimental results of this study verified the feasibility of the proposed system. The proposed system incurs a low computational cost (on hardware) for AI applications, and its controllable and programmable IoT devices can work independently to provide additional functions and services. The data stored in the proposed system can be secured and chained, and information related to certain data can be found and traced.

## Data Availability

The data presented in this study are available on request from the corresponding author. The data are not publicly available due to patent protection.
